# Supplementary material for: Increased Methylation of Brain-Derived Neurotrophic Factor (BDNF) Is Related to Emotionally Unstable Personality Disorder and Severity of Suicide Attempt in Women
Source: Cells. 2023 Jan 17;12(3):350. doi: 10.3390/cells12030350 (PMC9913473; doi:10.3390/cells12030350)
Supplement: Supplementary file 1 [file cells-12-00350-s001.zip › cells-2015453-supplementary.pdf]

Supplementary Figures

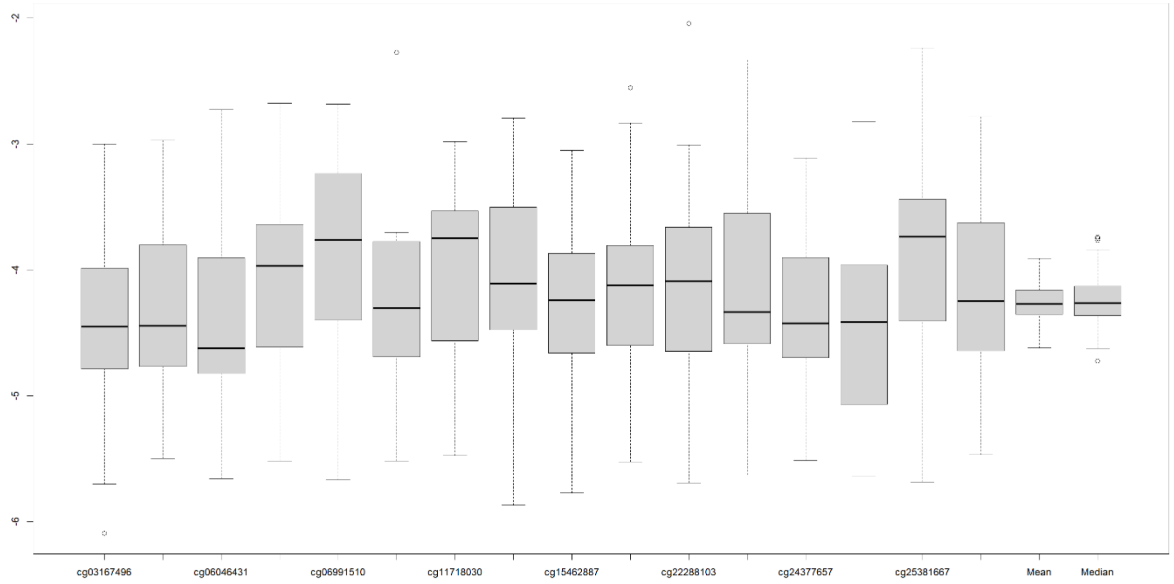

Supplementary Figure S1. Boxplots DNA methylation M-values for BDNF-coupled CpG-sites, their Median and Mean in the Discovery Group. Comparison of M-transformed methylation levels for independent BDNF-coupled CpG-sites, their median and mean in the Discovery group.

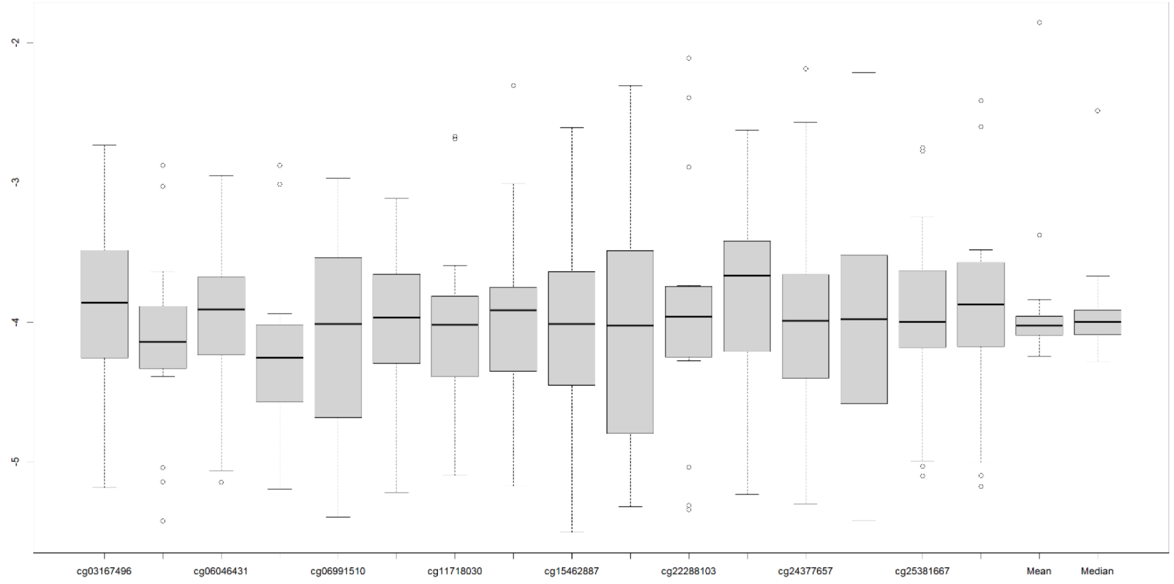

Supplementary Figure S2. Boxplots DNA methylation M-values for BDNF-coupled CpG-sites, their Median and Mean in the Validation Group. Comparison of M-transformed

methylation levels for independent BDNF-coupled CpG-sites, their median and mean in the Validation group.
